# Identification of intestinal mediators of *Caenorhabditis elegans* DBL-1/BMP immune signaling shaping gut microbiome composition

**DOI:** 10.1128/mbio.03703-24

**Published:** 2025-01-29

**Authors:** Dan Kim, Kenneth Trang, Barbara Pees, Siavash Karimzadegan, Rahul Bodkhe, Sabrina Hammond, Michael Shapira

**Affiliations:** 1 Department of Integrative Biology, University of California, Berkeley, Berkeley, California, USA; Centre d'Immunologie de Marseille-Luminy, Marseille, France; Rice University, Houston, Texas, USA

**Keywords:** *Caenorhabditis elegans*, gut microbiome, DBL-1/BMP signaling

## Abstract

The composition of the gut microbiome is determined by a complex interplay of diet, host genetics, microbe-microbe interactions, abiotic factors, and stochasticity. Previous studies have demonstrated the importance of host genetics in community assembly of the *Caenorhabditis elegans* gut microbiome and identified a central role for DBL-1/BMP immune signaling in determining the abundance of gut *Enterobacteriaceae*. However, the effects of DBL-1 signaling on gut bacteria were found to depend on its activation in extra-intestinal tissues, highlighting a gap in our understanding of the proximal factors that determine microbiome composition. In the present study, we used RNA-seq gene expression analysis of wildtype, *dbl-1* and *sma-3* mutants, and *dbl-1* over-expressors to identify candidate DBL-1/BMP targets that may mediate the pathway’s effects on gut commensals. Bacterial colonization experiments in mutants, or following RNAi-mediated knock-down of candidate genes specifically in the intestine, demonstrated their local contribution to intestinal control of *Enterobacteriaceae* abundance. Furthermore, epistasis analysis suggested that these contributions were downstream of the DBL-1 pathway, together suggesting that examined candidates were intestinal effectors and mediators of DBL-1 signaling, contributing to the shaping of gut microbiome composition.

**IMPORTANCE:**

Compared to the roles of diet, environmental availability, or lifestyle in determining gut microbiome composition, that of genetic factors is the least understood and often underestimated. The identification of intestinal effectors of distinct molecular functions that control enteric bacteria offers a glimpse into the genetic logic of microbiome control as well as a list of targets for future exploration of this logic.

## INTRODUCTION

Animals harbor large gut microbial communities (microbiomes) that play important roles in host health and fitness. The composition of these communities is shaped by various factors, including environmental microbial availability, diet, lifestyle, and host genetics (1). In recent years, a greater appreciation is emerging of the roles that host genetics play in the interactions between animals and microbes (2), but overall, host genetics remains less characterized compared with other determinants of gut microbiome composition. In humans, genome-wide association studies have revealed associations between gene variants and gut microbiome composition, including between variants of the LCT lactase gene and *Bifidobacteriaceae*, thought to be linked through lactose availability, or between *ABO* blood type variants and several different bacterial families, depending on the cohort (3). In turn, studies in mice comparing gut microbiome composition between wild-type mice and loss-of-function mutants revealed contributions of several innate immune-related genes to determining the composition of the gut microbiome (4–7). However, the role of host genes in determining microbiome composition is sometimes not immediately discernable in mouse mutants, requiring several generations to become evident, which in some cases was interpreted to be the result of community drift rather than the mutation itself, although in other cases such “drift” was subsequently shown to be indeed due to accumulating effects of candidate gene disruptions (8–10).

Invertebrate model organisms such as *Drosophila melanogaster* and *Caenorhabditis elegans* offer alternative models with greater genetic tractability and, similar to vertebrates, have demonstrated the importance of host immunity for controlling gut microbiome composition (11–14). Work in *Drosophila* revealed differential activation of immune mechanisms by pathogens or by non-pathogenic gut commensals, highlighting the ability of the innate immune system (which *Drosophila*, as all other invertebrates, solely rely on) to provide variable responses to maintain homeostasis and prevent collateral damage (11, 15). Work with age-synchronized populations of *C. elegans,* in turn, demonstrated how an age-dependent decline in a pathway of immune control was associated with age-dependent dysbiosis and the importance of a diverse gut community for preventing the detrimental consequences of this dysbiosis (16).

“Common garden” experiments, in which different *C. elegans* strains and related species were raised in identical compost microcosms, identified a significant contribution of host genetics to determining microbiome composition (17). Subsequent studies identified conserved regulatory pathways, including insulin/insulin-like (IIS) signaling (18, 19) and the DBL-1/BMP pathway (12), as contributing to shaping of the gut microbiome. DBL-1 signaling further came to the forefront as a mechanism that controls a specific subset of gut bacteria, which has the potential to cause detrimental effects when control was impaired (12). The DBL-1 ligand, a BMP-1 homolog, is primarily expressed in neurons (20), and upon secretion activates a broadly expressed heterodimer receptor, and downstream to it drives nuclear localization of transcriptional regulators SMA-2, -3, and -4 to activate gene expression (21). While DBL-1 signaling contributes both to larval development and to immunity, its effects on the gut microbiome were linked specifically to its immune contributions (12). Disruption of genes for any of the DBL-1 pathway’s components led to an expansion specifically of gut bacteria of the *Enterobacteriaceae* family, particularly of the genus *Enterobacter*. However, experiments attempting to rescue DBL-1 control in *sma-3* mutants, through tissue-specific *sma-3* expression, revealed that control over gut *Enterobacter* could not be achieved through intestinal *sma-3* expression and that, instead, expression from extra-intestinal tissues could restore control, suggesting that DBL-1 and SMA-3 signaling affected the gut microbiome cell non-autonomously, likely dependent on downstream activation of intestinal mediators (22).

Contributions of central regulatory pathways to shaping of the gut microbiome are large and, thus, easier to detect. Identifying smaller contributions of individual downstream effectors is more of a challenge. To identify intestinal effectors operating potentially downstream to DBL-1 signaling, we carried out RNA-seq analysis and subsequent functional characterization of candidate mediators, which led to the identification of several effectors with potential additive contributions to the control of *Enterobacteriaceae* gut colonization. The identified effectors describe what appears to be a gene network operating downstream to DBL-1 signaling, contributing to the shaping of the gut microbiome.

## RESULTS

### Altered gene expression downstream of DBL-1/BMP perturbations includes microbiome-modulated immune genes

**Fig 1:**
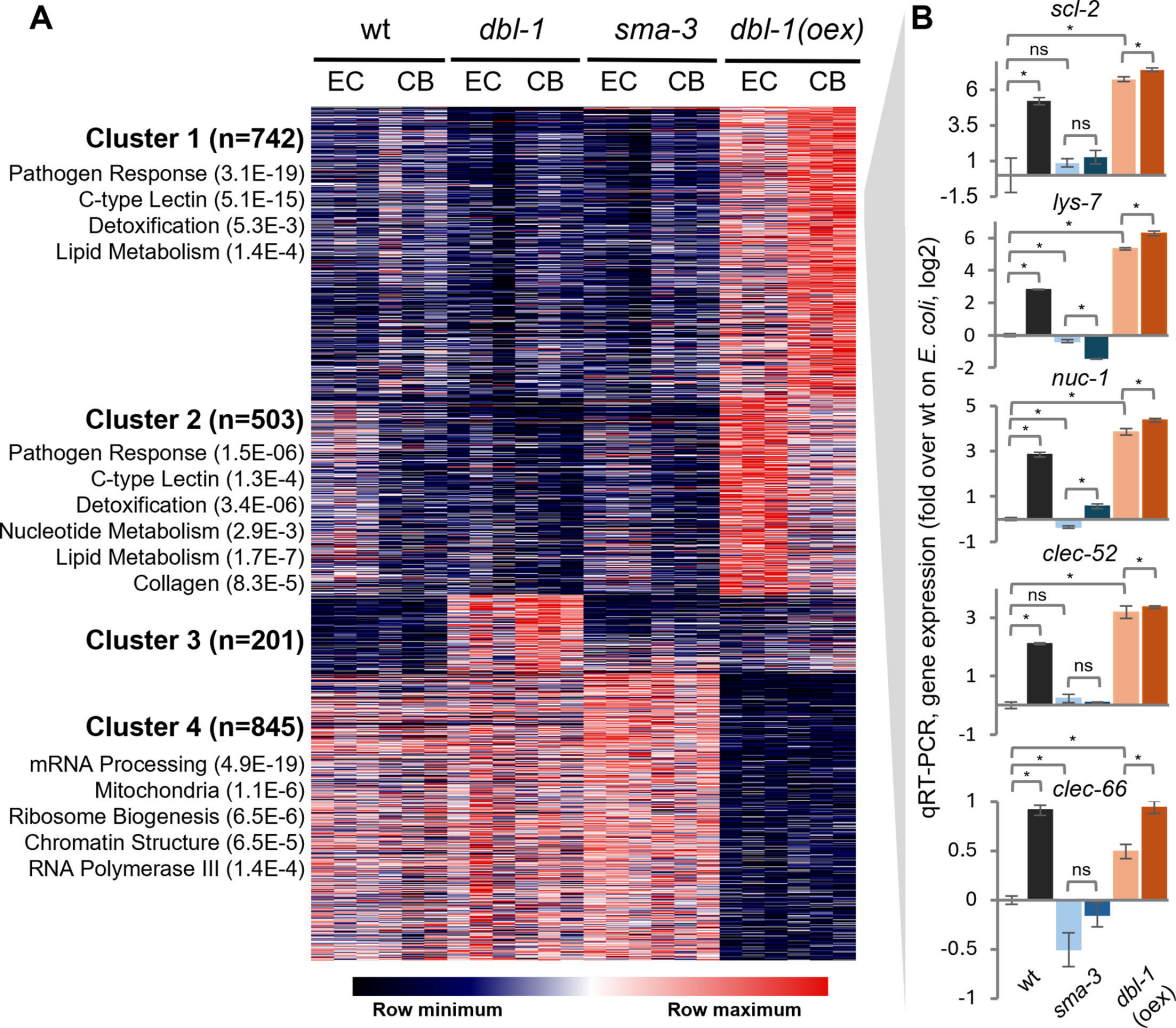
DBL-1/BMP-dependent gene expression. (**A**) Expression profiles of Sleuth-identified genes, differentially expressed (*P* < 0.005, BH-corrected, see the Materials and Methods) in wild-type and in designated mutant and transgenic strains raised on *E. coli* (EC) or on CeMbio (CB). Genes are *k*-means-clustered (with a number of genes for each cluster) and colored following median-centering for each gene to highlight patterns. Enriched gene annotations were identified using WormCat, with Bonferroni-corrected *P*-values. (**B**) qRT-PCR verification of expression patterns for selected genes of Cluster 1 in the designated strains; light and dark colors represent expression in worms raised on *E. coli* or CeMbio, respectively. Shown for each graph are averages of three measurements (*N* = 3) ± SDs. *P < 0.05, *t*-test.

To identify genes regulated by DBL-1/BMP signaling in the context of interactions with a complex microbial community, we performed RNA-seq analysis comparing gene expression in adult wild-type worms, *dbl-1* and *sma-3* mutants, and *dbl-1* over-expressing transgenics, raised either on non-colonizing *E. coli* or on the CeMbio community. Sleuth analysis identified 2,291 genes differentially expressed in DBL-1/BMP-perturbed strains (*q* < 0.005), divided between four clusters with distinct expression patterns (Fig. 1A; Data S1). Cluster 1 included 742 genes that were upregulated to varying extents on CeMbio, less so in either one of the two mutant strains, and much more in *dbl-1* over-expressing worms; Cluster 2 included 503 genes, which while also dependent for their basal expression on DBL-1 signaling (lower in mutants, higher in over-expressing animals), were repressed on CeMbio. Analysis of enriched annotations revealed enrichment for immune and stress response genes in both clusters, including C-type lectins and genes involved in detoxification, supporting the previously described roles of the DBL-1 pathway in immune regulation (12, 23). However, differences in gene composition between the two clusters were also apparent, with the CeMbio-upregulated genes of Cluster 1 showing a prominent enrichment for C-type lectins, while the CeMbio-downregulated genes of Cluster 2, showed more significant enrichment for detoxification genes, suggesting that DBL-1 signaling contributed differentially to the expression of different subsets of immune and stress genes. Cluster 1 further featured a significant enrichment for genes previously identified to be induced in response to two different complex communities (6 of 30 genes, *P* < 0.001, hypergeometric test, Table S1) (12). Cluster 4 was of additional interest, including 844 genes that were negatively regulated by DBL-1 signaling. Among them, enrichment was found for genes involved in house-keeping functions, such as mRNA processing (e.g., *prp-31*, *rnp-5*) and protein synthesis (e.g., *rps-21* and *rpl-21*), suggesting the involvement of DBL-1 signaling in negative regulation of growth and maintenance functions in adults in contrast to its better known positive contributions to cell growth in larvae (24).

Focusing on genes of Cluster 1—positively regulated by DBL-1 signaling and upregulated in response to CeMbio—we selected five*—scl-2*, *lys-7*, *clec-52, nuc-1,* and *clec-66,* for additional analyses. The first four of these were previously found to be up-regulated in worms raised on complex bacterial communities, but their functional significance remained unknown (12). All five are expressed primarily or exclusively in the intestine (25–29) and contain signal peptides targeting their secretion (30), suggesting that they could interact directly with gut bacteria. The five selected provide a mix of different functions, some known, others only presumed, and all uncharacterized with respect to their involvement in controlling the microbiome. *scl-2* encodes a yet uncharacterized protein homologous to mammalian cysteine-rich secreted proteins and peptidase inhibitors, which are best characterized for their ability to coat sperm cells to facilitate fertilization (31); *lys-7* encodes a lysozyme with documented roles in anti-bacterial defense; *clec-52* (ortholog of human Reg3α) and *clec-66* encode C-type lectins, thought to bind bacterial surface saccharides (32, 33); and lastly, *nuc-1* encodes a nuclease that degrades apoptotic DNA (34), which was additionally suggested to digest bacterial DNA in the intestine (27).

qRT-PCR measurements in samples of a separate experiment with wild-type animals, *sma-3* mutants (which, among different DBL-1 signaling mutants show the strongest bacterial blooms), and *dbl-1* over-expressors, confirmed the expression patterns first observed for the five candidates with RNA-seq. This experiment demonstrated the upregulation of the tested genes in response to CeMbio in both wildtype and *dbl-1* over-expressors compared to *E. coli*, and loss of this upregulation in *sma-3* mutants, with *nuc-1* somewhat of an exception, showing slight upregulation on CeMbio in *sma-3* mutants, but not to the extent observed in wild-type animals (Fig. 1B). This supported the hypothesis that the five genes were regulated downstream of DBL-1 signaling. At the same time, while basal expression of all five genes (on *E. coli*) was elevated in *dbl-1* over-expressors compared to wild-type animals, it was largely unaffected by *sma-3* disruption, suggesting additional regulatory inputs, in agreement with previous reports (35–37).

### Intestinal contributions of putative DBL-1 targets to *Enterobacter* gut colonization

**Fig 2:**
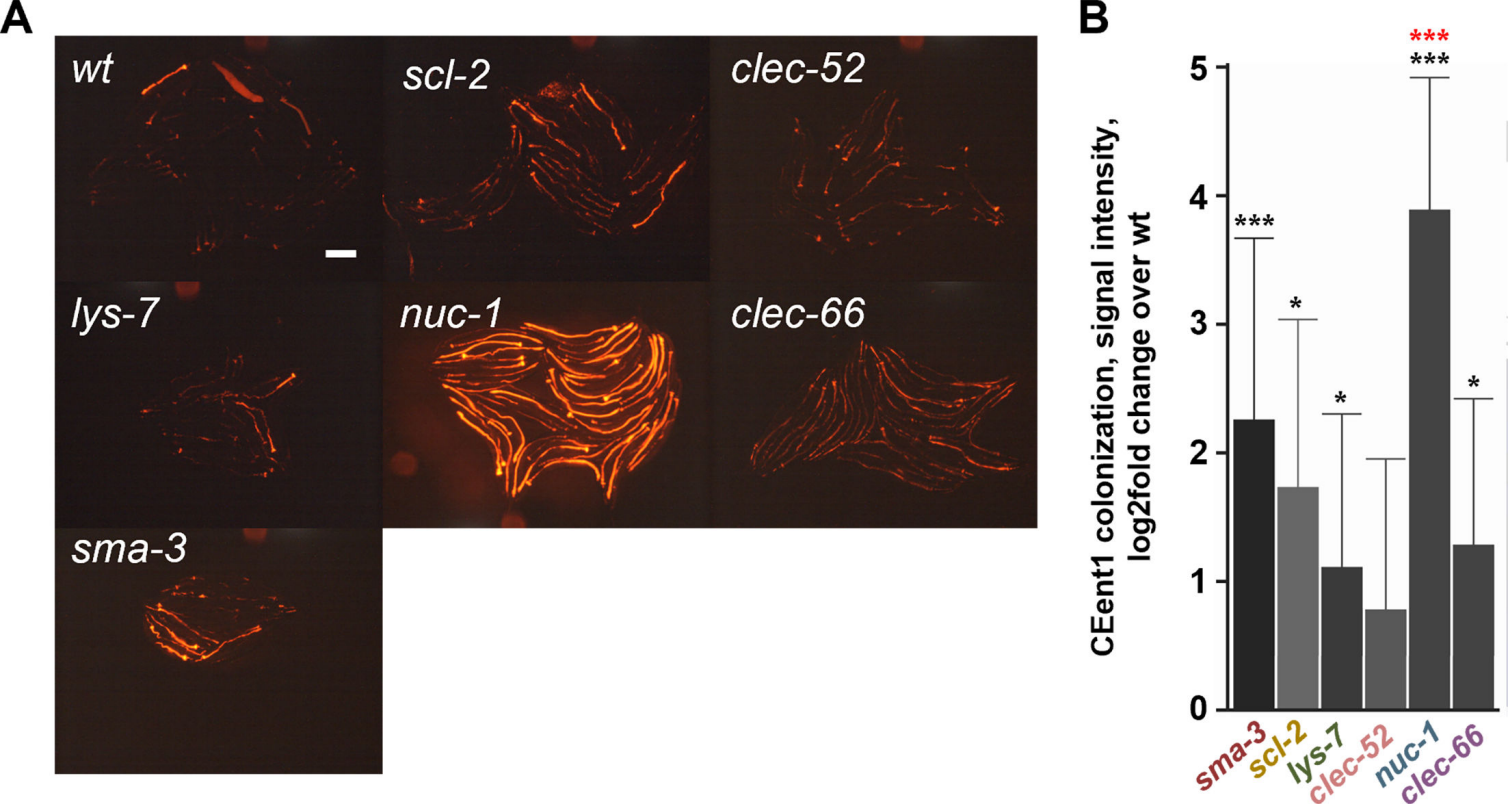
Disruption of selected putative DBL-1 target genes increases *Enterobacter* colonization. (**A**) Representative images of DBL-1/BMP effector mutant strains grown on dsRed-CEent1 bacteria, compared to wild type, recorded 1-day after L4. Scale bar, 200 µm. (**B**) Quantification of signal intensity in worms as in A. Shown are averages ± SDs for three experiments per strain, each with *N* = 20–46 worms (except *nuc-1* mutants*,* which were included in two experiments only). *P < 0.05, ***P < 0.001, *t*-test compared to wild-type animals (black asterisks) or *sma-3* mutants (red asterisks).

Previous work has used a fluorescently(dsRed)-tagged derivative of a *C. elegans* commensal, *Enterobacter hormaechei* strain CEent1, to demonstrate the role of DBL-1 signaling in controlling gut abundance of bacteria of the *Enterobacteriaceae* family (12, 16). Using the tagged strain, we examined whether the presently identified putative DBL-1 targets contributed to gut bacterial control, assessing gut colonization in mutants for the five DBL-1 targets. When raised on monocultures of CEent1-dsRed, all five mutants showed increased colonization compared to wild-type worms (although for *clec-52,* the increase was not statistically significant; Fig. 2). For the most part, the extent of increased colonization in the mutants was lower than in *sma-3* mutants, except for *nuc-1* mutants, which showed colonization significantly greater than that seen in *sma-3* mutants.

**Fig 3:**
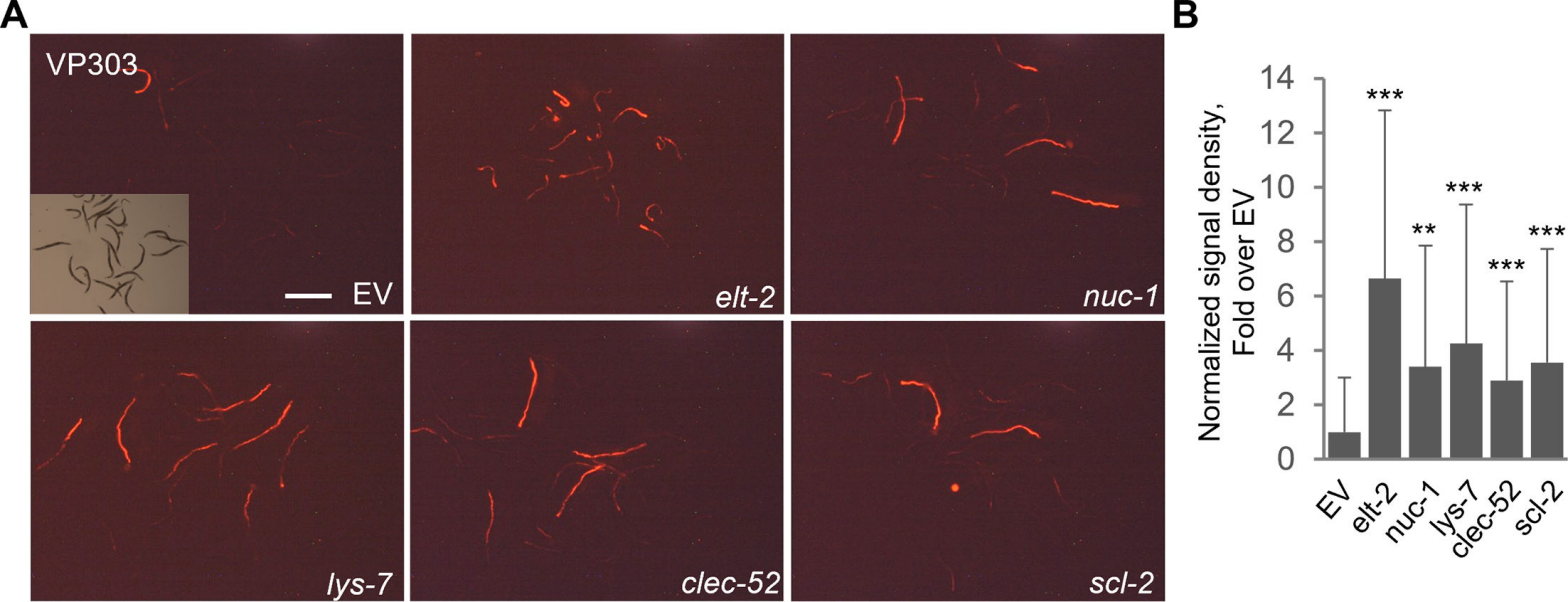
Intestinal RNAi-mediated silencing of putative DBL-1 targets increases *Enterobacter* colonization. (**A**) Representative images of transgenic VP303 worms raised from egg to L4 on RNAi-expressing clones targeting effector candidates [or with empty vector (EV) as control] and shifted to colonizing CEent-1-dsRed bacteria for 2 days. Scale bar = 500 µm. (Inset) A visible-light image of the same field as in the fluorescent image showing also the uncolonized worms not seen in the fluorescent image. (**B**) Quantification of fluorescent signal in images as in A; signal density normalized by body size. Shown are average ± standard deviations for *N* = 23–41 worms/group, *P < 0.05, **P < 0.01, ***P < 0.001, *t*-test, compared to empty vector (EV).

As described above, previous reports suggested that the examined genes were expressed primarily in the intestine. However, increased CEent1 colonization in mutants for these genes could still be due to lesser known extra-intestinal expression. To examine whether intestinal expression of the putative DBL-1 targets was required for their effects on gut colonization, we employed RNAi-mediated expression knock-down, in which worms are raised on *E. coli* clones expressing double-stranded RNAi targeting genes of interest. Gene knock-down was carried out in worms of the VP303 strain, in which RDE-1 activity, which is required for RNAi processing, was disrupted, but transgenically-rescued by intestine-specific expression of wildtype *rde-1*, enabling intestine-specific knock-down. Worms raised on *E. coli* containing an empty RNAi vector (EV) served as negative control, and worms raised on *E. coli* expressing dsRNA targeting *elt-2* were used as positive control, ELT-2 being a master regulator of intestinal immune gene expression (38). No RNAi clone was available for *clec-66*. This experiment revealed that intestine-specific knock-down of each of the four examined genes was sufficient to increase CEent1-dsRed gut colonization (Fig. 3). Overall, the effects of RNAi-mediated knock-down were comparable to those seen in the respective mutants, suggesting that effects on gut colonization were attributed to candidate genes intestinal expression. The exception was again *nuc-1*, which in knock-down experiments showed a smaller effect on colonization compared to the respective mutant. This might be due to incomplete knock-down associated with the available *nuc-1* RNAi clone but, alternatively, may suggest that *nuc-1* contributions to intestinal control of bacterial colonization included both local as well as extra-intestinal inputs.

**Fig 4:**
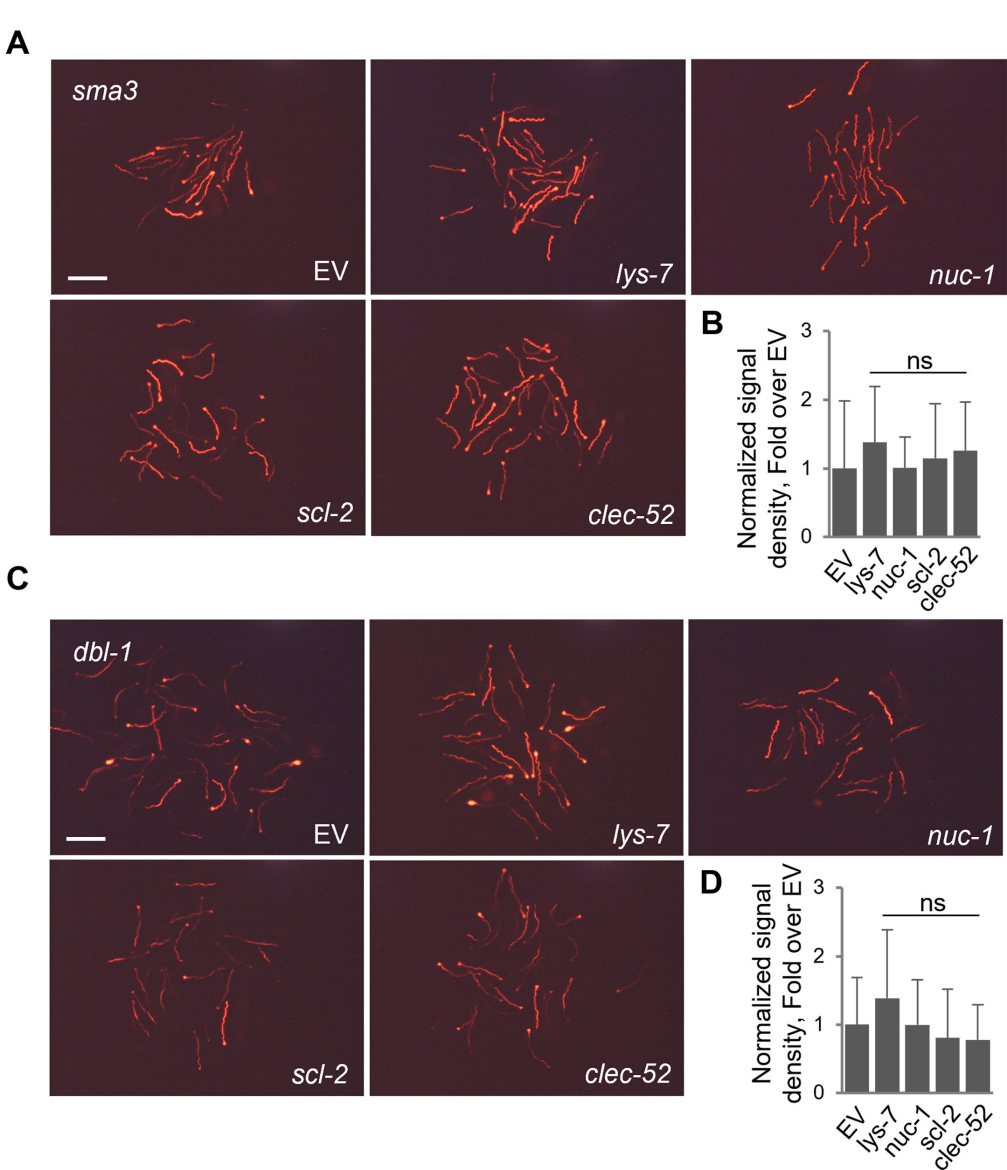
Knock-down of putative DBL-1 targets in DBL-1 mutants has no added effect on gut colonization. Images of CEent1-dsRed colonization in (**A**) *sma-3* or (**C**) *dbl-1* mutants raised from egg to L4 on RNAi targeting designated genes (bottom of images) and then shifted to CEent-1-dsRed bacteria for 2 days. Scale bar, 500 µm. Quantification of fluorescent signal in images, normalized to body size, is shown in (**B**) and (**D**), respectively. Graphs show average ± standard deviations for *N* = 24–35 (*sma-3* mutants), or *N* = 23–34 worms (*dbl-1* mutants). ns, *P* > 0.05, *t*-test compared to empty vector (EV).

RNAi-mediated knock-down was further used to assess whether the examined genes contributed to colonization-control downstream of DBL-1 signaling or independently of it. To this end, the expression of putative DBL-1 targets was knocked down in *sma-3* or *dbl-1* mutants and CEent1-dsRed colonization was assessed to test whether knock-down led to increased colonization on top of that seen in the mutants alone. With both mutants, we found no added effects with any of the RNAi clones (Fig. 4). While strong colonization in *sma-3* mutants prior to candidate gene knock-down may impede observing additive effects (Fig. 4A and B), weaker colonization in *dbl-1* mutants should have left enough range to observe added effects if those existed, yet, none was observed (Fig. 4C and D). Together, the results shown in Fig. 3 and 4 support the notion that the examined genes encoded intestinal effectors that mediated DBL-1/BMP-dependent control of gut colonization.

### Identified effectors are important for *Enterobacteriaceae* control in the context of a complex community

**Fig 5:**
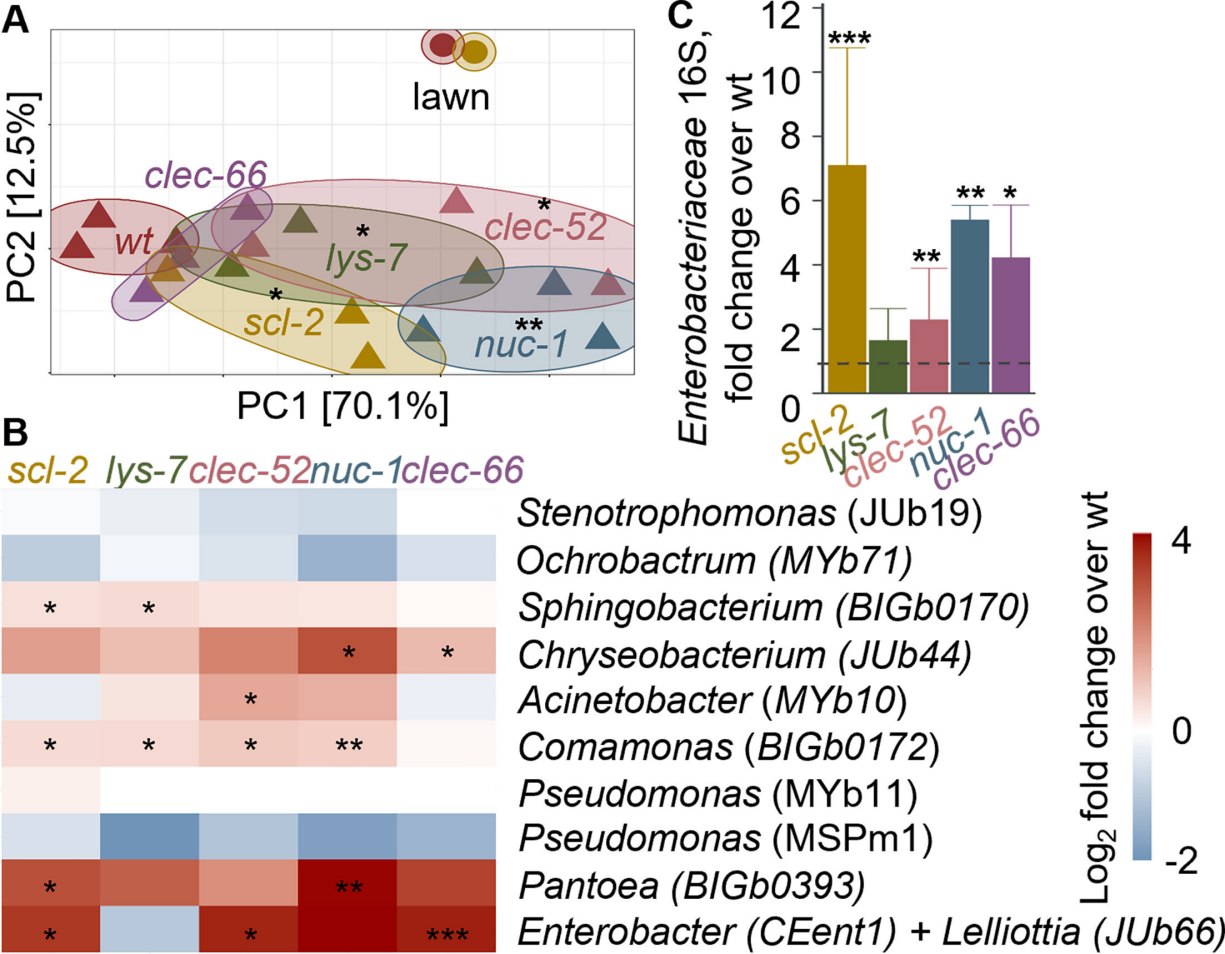
Disruption of intestinal DBL-1 targets alters gut microbiome composition. (**A**) PCoA based on weighted UniFrac distances highlighting differences in microbiome composition (analyzed by 16S NGS) between wildtype and mutant worms in one experiment, analyzed for each strain in three independent populations; *P < 0.05, **P < 0.01, UniFrac regression-based kernel association test with small-sample size correction. (**B**) Data from A, highlighting relative abundances of CeMbio members in tested mutants, shown as fold over wildtype; *P < 0.05, **P < 0.01, ***P < 0.001, *t*-test, compared to wildtype. (**C**) *Enterobacteriaceae* relative abundance in designated mutants, including results from several independent experiments as the one presented in A (four independent experiments for *scl-2* and *clec-52* mutants*,* two for *nuc-1* and *clec-66,* and one for *lys-*7), each experiment performed with 3–5 worm populations (*N* = 250–500 worms/population). Values are shown as fold over wildtype, *P < 0.05, **P < 0.01, ***P < 0.001, *t*-test compared to wildtype.

**Fig 6:**
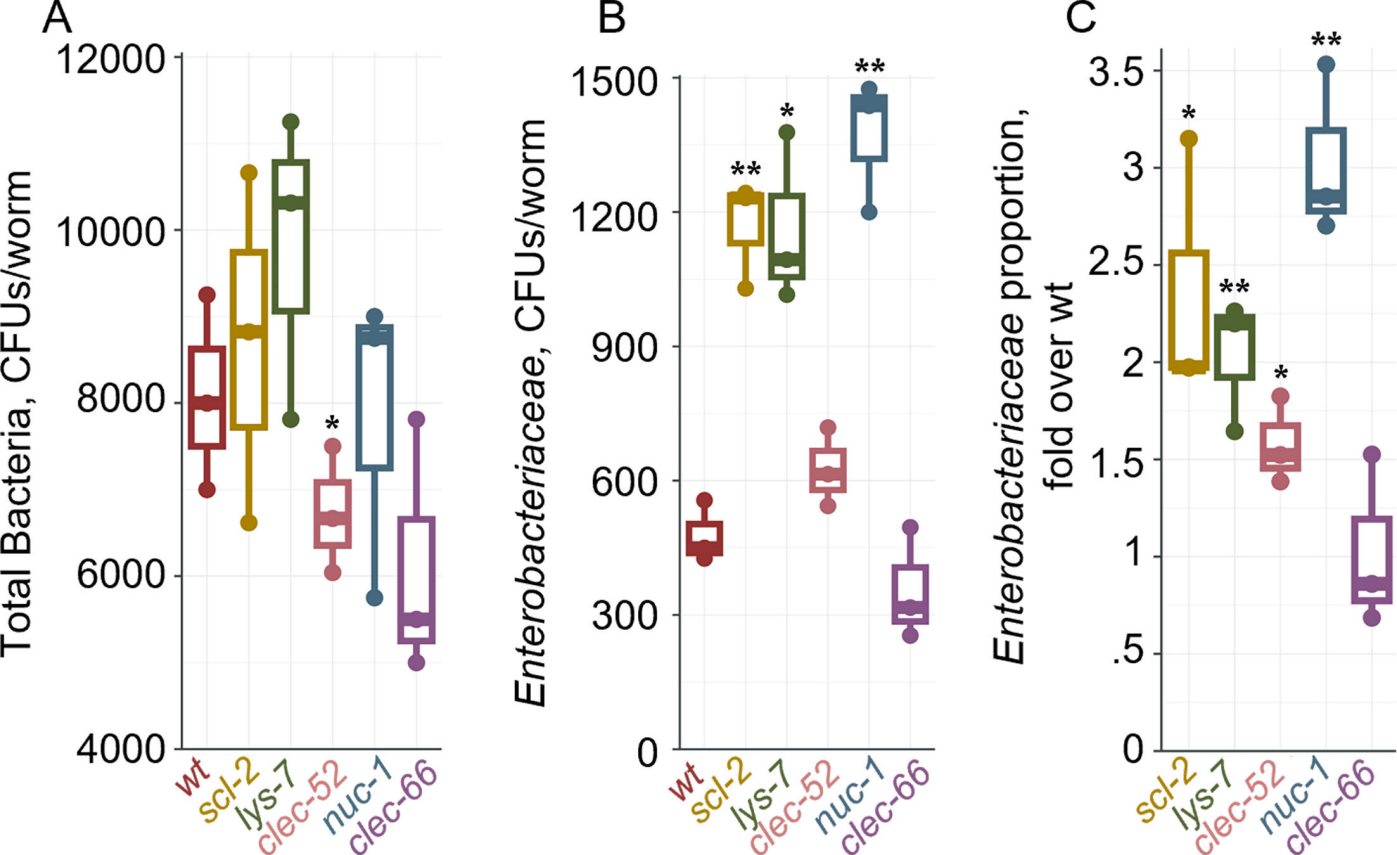
Disruption of intestinal DBL-1 targets increases gut *Enterobacteriaceae* load. (**A**) Total bacterial load, represented by CFU counts on LB plates. (**B**) *Enterobacteriaceae* gut load, counted on selective VRBG media. (**C**) *Enterobacteriaceae* proportion out of the total bacterial load relative to wildtype calculated from data in A and B. Shown for each graph are median and interquartile from three replicate populations (*N* = 68–200 worms/group), *P < 0.05, **P < 0.01, ***P < 0.001, *t*-test compared to wild-type animals.

To test how disruption of the identified DBL-1 targets/effectors may affect a more complex gut community rather than a single colonizer, we raised wildtype and mutant worms on the CeMbio community of 12 worm commensal strains (see Materials and Methods) and analyzed their gut microbiome composition using 16S sequencing (of the V4 region). This analysis identified significant differences between wild-type animals and most mutants, excluding *clec-66* (Fig. 5A). Gut microbiomes assembled from CeMbio tend to be dominated by two strains*—Ochrobactrum vermis* (MYb71) and *Stenotrophomonas indicatrix* (JUb19)—contributing 70%–80% of total bacterial abundance (39), and this dominance was maintained in the examined mutants (Data S2). However, relative abundance of *Enterobacteriaceae* strains—*E. hormaechei* (CEent1) and *Lelliottia amnigena* (JUb66), which cannot be distinguished based on V4 16S sequencing, reproducibly increased in four out of five mutants (excluding *lys-7*), extending the previously described role of DBL-1 signaling in controlling bacteria of the *Enterobacteriaceae* family to its putative downstream mediators (Fig. 5B and C). Additional experiments were performed to complement the sequencing analysis of gut microbiome composition, using CFU counts of gut bacteria isolated from wildtype and mutant worms raised on CeMbio. Samples were split between rich media and *Enterobacteriaceae*-selective VRBG media plates, to assess total bacterial load, or *Enterobacteriaceae* load, respectively. While total bacterial load did not change significantly in most of the mutants compared to wild-type animals (Fig. 6A), the overall gut abundance of *Enterobacteriaceae* increased significantly in *scl-2*, *lys-7*, and *nuc-1* mutants, and its proportion within the gut community increased in these three as well as in *clec-52* mutants (Fig. 6B and C). Together, the results from experiments using different methodologies support the involvement of *scl-2* and *nuc-1* in controlling *Enterobacteriaceae* gut abundance in both monocultures and in the context of a complex community. The results further support the involvement of *lys-7*, *clec-52,* and *clec-66* in this control albeit with smaller and more variable contributions.

## DISCUSSION

Previous identification of DBL-1/BMP immune signaling as a factor determining gut microbiome composition, specifically controlling the abundance of *Enterobacteriaceae*, raised the question of what mediated its effects on gut bacteria. DBL-1-dependent control was deemed to involve several regulatory levels, as its effects depended on the activation of transcriptional regulators in extra-intestinal tissues (12, 22). The results described here begin to fill in this gap by identifying several mediators, which could directly interact with gut bacteria to control their abundance. The five mediator genes, *scl-2*, *lys-7*, *clec-52*, *clec-66*, and *nuc-1,* are positively regulated by DBL-1 signaling, and their disruption in wild-type animals led to increases in gut relative abundance of bacteria of the *Enterobacteriaceae* family but was redundant when carried out in DBL-1 signaling mutants, supporting their contributions to gut bacterial control downstream of DBL-1 signaling. Importantly, intestinal-specific knock-down of their expression resulted in increased *Enterobacter* colonization that was comparable (for four out of the five, see discussion of *nuc-1* below) to that seen in mutants, supporting intestinal function. Given the secretion signal peptides that are part of the five gene-products (30), it is plausible that contributions of the five genes to bacterial control involve direct lumenal interactions with gut *Enterobacteriaceae*.

Disruptions of four of the five identified effectors caused increases in *Enterobacter* colonization that were smaller than those caused by disruption of their upstream regulator gene *sma-3*. These findings support the hypothesis that DBL-1 signaling controls *Enterobacter* colonization through a combination of downstream effectors, which contribute additively to bacterial control (such weak additive effects may further explain the less robust effects that some of the mutants had on colonization). Other regulatory pathways may induce the expression of other antimicrobial cocktails, partially overlapping in their composition to those regulated by DBL-1 and affecting non-*Enterobacteriaceae* gut bacteria. For example, insulin signaling (IIS), mediated by the DAF-16/FOXO transcription factor, was shown to control the abundance of bacteria of the genus *Ochrobactrum*, also common inhabitants of the worm gut (18). Through partially overlapping antimicrobial cocktails, a few regulatory pathways could differentially control gut microbes and shape microbiome composition. Several studies, primarily in *Drosophila*, demonstrated the contributions of different immune regulators to the abundance of different gut constituents (11). A recent study, also in *Drosophila*, nicely demonstrated differential control, describing specific effects of Diptericin A and B, two antimicrobial peptides regulated by the Imd immune pathway, on two distinct gut commensals (40). This observation further suggests that diversification of antimicrobial proteins may be driven not only by the need to fight pathogens but also by the need to control gut microbiome.

Of the five studied mediators, the lysozyme gene and the C-type lectin genes are known to be associated with responses to pathogenic bacteria (23, 41, 42). Our results extend their function to controlling non-pathogenic commensal bacteria. At least for one of these genes, *clec-52,* this involvement may be conserved, as enteric delivery of the human *clec-52* homolog Reg3A in mice was shown to alter gut microbiome composition and to reduce colitis (43). In contrast, *scl-2* and *nuc-1* are not typically associated with immune responses but are consistently found to be upregulated in worms exposed to complex microbial communities (12), supporting their involvement in host-microbiome interactions. Interestingly, it was these last two that showed the strongest effects on gut *Enterobacteriaceae* abundance. In particular, *nuc-1* mutants showed strong effects on gut *Enterobacteriaceae* abundance both with the CeMbio community and with *Enterobacter* monocultures in the latter showing larger effects than those seen in *sma-3* mutants. *nuc-1* further seems to have both intestinal and extra-intestinal contributions to *Enterobacter* control, suggesting that it may function in the intestine downstream to DBL-1 signaling but may have also extra-intestinal contributions that are independent of SMA-3/DBL-1 signaling and are additive to it. *nuc-1* encodes a DNase II homolog and was suggested to be secreted and then taken up by apoptotic cells, where it helped in degrading their DNA (34). It was also reported to be involved in the degradation of bacterial DNA in the intestinal lumen (27), yet, none of these reported activities could explain how it might affect live intact gut bacteria to control their abundance. Another study suggested that *nuc-1* disruption in the germline upregulated antimicrobial peptides (44). Such an effect, originating outside of the intestine, may help explain the higher gut abundance of *Enterobacter* seen in *nuc-1* mutants compared to animals with intestinal-specific disruption. Nevertheless, increased expression of antimicrobial peptides does not fit the increase we observed in bacterial abundance.

While the mechanisms underlying the effects of the identified intestinal mediators on the gut microbiome remain to be investigated, the results presented here describe a new layer in host control over its gut bacteria and expand our understanding of the role of the *C. elegans* DBL-1 signaling in such control to describe an underlying gene network that mediates its effects on the worm gut microbiome.

## MATERIALS AND METHODS

### Worm strains

Worm strains used in this study included the N2 wild-type strain, *dbl-1(nk3), sma-3(e491*), and the *dbl-1* overexpressing strain BW1940[*dbl-1p::dbl-1;sur-5::gfp*] (20), *lys-7(ok1384*), *nuc-1(e1392*), and *clec-66(ok2230*), all obtained from the *Caenorhabditis* Genome Center (CGC), and *clec-52(tm8126*) and *scl-2(tm2428),* obtained from the National Bioresource Project (45). The VP303 strain (*rde-1(ne219);kbIs7* [*nhx-2p::rde-1 + rol-6(su1006*)]) was gratefully received from the Dillin lab. Worms were raised on standard nematode growth medium (NGM), with bacteria as food or as colonizers. Time for worm collection was according to developmental stage to account for slower development rate for some of the mutants.

### Bacterial strains and communities

Bacterial strains and communities included the non-colonizing *E. coli* strain OP50, used as food and as control, CeMbio (39), a defined community of *C. elegans* gut commensals consisting of 12 characterized strains selected to represent the core *C. elegans* gut microbiome, and CEent1-dsRed, a fluorescently-tagged derivative of the *Enterobacter hormachei* strain CEent1, a member of the CeMbio community (16). CeMbio strains were raised as previously described (39), normalized based on optical density, mixed in equal proportions and seeded on NGM plates.

### RNA-seq

Germ-free L1 larvae obtained from gravid worms by bleaching (three independent populations per worm strain) were raised at 25°C on NGM plates seeded with CeMbio as described above. Early gravid worms were rinsed off plates with M9 including 0.025% Triton, washed 5 times to get rid of offspring and bacteria, mixed with TRIzol Reagent (Invitrogen; Waltham, USA), snap-frozen in liquid nitrogen, taken through 5–7 thaw-freeze cycles to break them open, and kept at −80°C until use. RNA isolation was performed using the NucleoSpin RNA purification kit, manual protocol 5.2 (Macherey-Nagel; Düren, Germany).

Sequencing libraries were prepared from total RNA using the TruSeq RNA Library Kit v2 (Illumina; San Diego, USA), with indexed adaptors for multiplex sequencing, assessed for quality on an Agilent Bio-analyzer (Agilent; Santa Clara, USA) and submitted for 100 bp paired-end sequencing on a NovaSeq 6000 at the QB3 Genomic Sequencing Laboratory (UC Berkeley, Berkeley, CA; RRID:SCR_022170). Raw reads obtained were pre-processed with *fastp* (46) and pseudo-aligned to the WormBase transcriptome version WS235 using *kallisto* (47). Transcript counts were then normalized with *Sleuth* (48) and analyzed to identify genes differentially expressed between worm strains and bacterial treatment using the likelihood ratio test. Heatmaps following *k*-means clustering (*k* = 4) were generated with *Morpheus* (https://software.broadinstitute.org/morpheus) and gene set enrichment analyses were performed using WormCat (49).

### Quantitative (q)RT-PCR

(q)RT-PCR measurements were performed on RNA extracted from worms raised at 20°C, as described above. mRNA was reverse transcribed with the iScript Reverse Transcription Supermix (BioRad, Hercules, USA), and cDNA was used for amplification using the SsoAdvanced Universal SYBR Green Supermix (BioRad, Hercules, USA) on an Applied Biosystems StepOnePlus cycler (Waltham, USA). Ct values obtained in the amplification of specific mRNAs were normalized to those obtained by the amplification of three conserved *C. elegans* actin genes with the pan-actin primer pair (41).

Primers used included:

*scl-2*: F: 5′-GATTTCGCCCACGCCATTTG-3′; R: 5′-ACTCAGAAATCGCCGGGAAC-3′

*lys-7*: F 5′-TTGCAGTACTCTGCCATTCG-3′; R: 5′-GCACAATAACCCGCTTGTTT-3′

*clec-52*: F: 5′-AGCCAAATCTCCTCCATCAGC-3′;

R: 5′-GATCAACCGCCTGTATGCAAC-3′

*nuc-1*: F: 5′-CCTGGAAGATGGTCTTGTCA-3′;

R: 5′-GGGAACTTTGACTCCTTCTGC-3′

*clec-66*: F: 5′-GCAGAAGGCGGTTTTGGC-3′; R: 5′-GCGGCGAATTTAGTCATGGC-3′

PanActin: F: 5′- TCGGTATGGGACAGAAGGAC-3′;

R: 5′-CATCCCATGTGGTGACGATA-3′

### RNAi-mediated gene expression knock-down

RNAi-mediated gene expression knock-down was carried out using standard *C. elegans* protocols (50). Wild-type or mutant worms were raised from egg to L4 at 25°C on NGM plates supplemented with 0.1 mg/mL Ampicillin and 1 mM IPTG (to induce dsRNA expression), seeded with concentrated *E. coli* strain HT115 clones from either the Ahringer RNAi library (50)*—nuc-1*, *lys-7*, and *elt-2*, or of the Open Biosystems library (51)*—scl-2* and *clec-52*. Worms were then washed with M9 minimal medium three times and transferred to NGM plates with CEent1-dsRed to allow colonization and subsequent imaging.

### DNA extraction for gut microbiome analysis

Early gravid worms raised at 20°C on NGM plates with CeMbio (three independent populations per worm strain) were washed off plates, washed five times with M9 + 0.025% Triton, paralyzed with levamisole to seal the intestine, surface sterilized with bleach as described elsewhere, and kept at 4°C until use. DNA was extracted using the Qiagen DNeasy PowerSoil Pro Kit, with modifications as described elsewhere.

### 16S rRNA gene sequencing

16S rRNA gene sequencing of the amplicon libraries of the V4 variable region generated with primers 515F and 806R containing Illumina overhang adapter sequences according to the manufacturer instructions, with slight cycling modifications described elsewhere. Dual indices and Illumina sequencing adapters were added using the Nextera XT Index Kit. Sequencing was performed on an Illumina MiniSeq.

Demultiplexed forward and reverse sequences were filtered for quality, resulting in roughly 11,000 reads per sample, and assigned amplicon sequence variants (ASVs) with DADA2 and *DECIPHER*. Taxonomy assignments for ASVs were obtained based on a custom database with 16S sequences of the 12 CeMbio strains, and counts were normalized for the different 16S gene copy number of the different strains. Microbiome analyses were performed in R using *phyloseq*, *phangorn*, and *vegan* to calculate UniFrac distances for Principle Component Analysis; and MiRKAT, for statistical evaluation of differences between microbiomes.

### CFU counts

CFU counts of gut commensals were evaluated in worms raised, harvested, and surface sterilized as described above. Gut bacteria were released from worms by vortexing together with zirconium beads until degradation could be confirmed using a light microscope. Serially diluted worm lysates were plated on either rich LB media or *Enterobacteriaceae*-selective media (Violet Red Bile Glucose, VRBG; Difco Becton Dickinson) and incubated at 28°C for 24 h before counting colonies. *Pseudomonas* species can also grow on VRBG, but colonies are distinguishable from the *Enterobacteriaceae* of interest.

### Fluorescence imaging

Fluorescence imaging was employed to follow worm colonization by *E. hormachei* CEent1, using the CEent1-dsRed derivative. Worms were raised from the L1 stage on a lawn of CEent-1-dsRed at 20°C. Following approximately 3 days, early gravid worms were washed off plates, washed three times with M9, and imaged. Fluorescent images were captured using a Leica MZ16F equipped with a QImaging MicroPublisher 5.0 camera and fluorescent signal of colonizing bacteria was quantified on the Fiji plugin of ImageJ v2.10/1.53c as previously described, producing background-subtracted average intensity for each worm.

## Data Availability

Raw RNA-seq data and kallisto output files have been deposited in GEO with accession number GSE186653; the associated computational pipeline is available online at https://github.com/rahulnccs/TGF-beta_RNAseq_Analysis. 16S sequencing data are available in the NCBI Sequence Read Archive (Bioproject ID PRJNA1031602), with the computational pipeline available at https://github.com/kennytrang/DBL-1_Mediators.

## SUPPLEMENTAL MATERIAL

Table S1 mbio.03703-24-s0001.xlsx Data from RNA-sequencing analysis.

Table S2 mbio.03703-24-s0002.xlsx Data from next-generation 16S sequencing experiment.

ASM does not own the copyrights to Supplemental Material that may be linked to, or accessed through, an article. The authors have granted ASM a non-exclusive, world-wide license to publish the Supplemental Material files. Please contact the corresponding author directly for reuse.
